# “Green informed consent” in the classroom, clinic, and consultation room

**DOI:** 10.1007/s11019-023-10163-x

**Published:** 2023-08-16

**Authors:** Cristina Richie

**Affiliations:** Ethics of Technology, Edinburgh Futures Institute, The University of Edinburgh, Edinburgh, Scotland

**Keywords:** Green informed consent, Environmental bioethics, Health care ethics, Medical education, Professionalism

## Abstract

The carbon emissions of global health care activities make up 4–5% of total world emissions, placing it on par with the food sector. Carbon emissions are particularly relevant for health care because of climate change health hazards. Doctors and health care professionals must connect their health care delivery with carbon emissions and minimize resource use when possible as a part of their obligation to do no harm. Given that reducing carbon is a global ethical priority, the informed consent process in health care delivery must change. I argue that the expanded role of bioethicists in this climate crisis is to promote and support “green informed consent:” the sharing of climate information with patients, offering options for lower-carbon health care, and accepting the patient’s right to decline treatments which are deemed too carbon intensive for their values.

Greenhouse gas emissions like carbon dioxide (CO_2_) contribute to climate change. The safe amount of carbon in the atmosphere—350ppm—has been surpassed (Hansen et al. 2008). Numerous world organizations, legislative bodies, and economic sectors have explored carbon limitation, including the health care industry (National Health Services 2009). The carbon emissions of global health care constitute 4–5% of total world emissions (Karliner et al. 2019), making it as carbon-intensive as the food sector (Pichler et al. 2019). Health care carbon comes from a variety of sources and some system, like the National Health Services in the UK, have already enacted legally binding carbon reduction measures. However, these tackle carbon which any business is responsible for, such as carbon from commuting to and from work, the emissions from energy use in building, and the food served in the cafeteria (National Health Services 2009). These are important steps towards greening the industry, but they simply do not go far enough. Health care delivery, that is, hospital care and physician and clinical services, are the two largest carbon contributors to health care carbon—exceeding even structures like buildings (Eckelman and Sherman 2016).

Moreover, carbon emissions of health care delivery are particularly relevant for the healing arts because of climate change health hazards. Climate change health hazards include medical burdens and deaths related to extreme heat, outdoor air quality, flooding, vector-borne infection, respiratory disease, and water- and food-related infection (World Health Organization 2009). The UK alone has thousands of hospital admissions and deaths each year attributable to climate change (Walkeden et al. 2022). These are not only financially costly but, of course, devastating to individuals and families.

Treating those affected by climate change health hazards also cause more carbon through influxes in hospital admissions and follow up medical care (Knowlton et al. 2011). Even as those being treated for climate change health hazards are cured, their treatments release more carbon, locking health care into a self-destructive cycle whereby medical care causes medical needs. Hence, health care has a special interest in carbon reduction, not only as a matter of international priority, but also as a commitment to health. There is a palpable conflict between doctors who participate in high-carbon vocations in many parts of the world—thus harming not only their own patients, but also other people, themselves not excluded—and the mission to prevent disease and do no harm.

Given that reducing carbon is a global ethical priority, the informed consent process in health care delivery must change. This article will argue that the expanded role of clinical ethics in this climate crisis includes promotion and support of “green informed consent:” the sharing of climate information with patients, offering options for lower-carbon health care, and accepting the patient’s right to decline treatments which are deemed to be too carbon intensive for the patient’s own values (Richie 2019). To build this argument, I will first sketch a basic framework of “green informed consent,” noting that, as with other aspects of informed consent, imposition of the health care provider’s values is never appropriate. I will then explain how green informed consent would fit into three important domains of health care, dubbed the Classroom, Clinic, and Consultation Room. In the classroom, green informed consent fits within already existing curriculum on medical burdens (i.e., how climate affects health) and the consenting process. In the clinic, green informed consent can be supported by professional development and continuing education of health care professionals and administrators so they can inform patients about the health care impact of treatments, when appropriate. During clinical ethics consultations, the environmental impact of health care/planetary health may be weighted as one of the “contextual features” of ethics. The conclusion will reiterate that green informed consent is just one tool that may be used to green health care generally and society more broadly.

## Green informed consent

Informed consent is a medical decision-making process whereby health care professionals determine appropriate medical treatments, provide clinically indicated options and alternatives of treatments to the patient, and explore the patients’ values, desires, and concerns related to the treatment and outcomes. For instance, if a man comes to a clinic for contraception, the health care provider may offer sterilization, abstinence, or male condoms, explaining the failure rates and side effects of each. The man can then voice his preferences. According to traditional Western biomedical ethics, if the treatment is clinically indicated and within the competencies of the health care provider, the choice of the patient should be respected by providing the desired care. Informed consent is based on the biomedical principle of respect for autonomy, which maintains that capable adults are able to make their own medical decisions when they have proper information.

Green informed consent, like informed consent, has a number of stages: (1) after medical diagnostics, the health care professional and patient (and/or surrogate decision maker) (2) have a conversation which information about the diagnosis and possible medical interventions are offered to the patient, based on clinical need. The health care professional explains the options, the benefits, and drawbacks of each. (3) The health care professional gathers information from the patient about the patient’s clinical and personal values. (4) The health care professional negotiates or discusses these options with the patient and comes to a clinical plan. (5) The plan is enacted (Faden and Beauchamp 1986). Each of these present opportunities and barriers to green informed consent.

First, green informed consent assumes that there is a clinically necessary procedure. Health care carbon reduction should be comprehensive and target first those medical services which are neither urgent, nor life saving, nor life-extending for reduction in number or kind before addressing the consent process in urgent, life-saving, and life-extending medicine. However, due to the interest and importance of sustainability, even luxury and elective medical services—such as in-vitro fertilization—have information on carbon and environmental impact of treatments. For instance,IVF Meeting, an educational and collaborative organization for in-vitro fertilization clinics—offers education and information the environmental impact of fertility treatments (IVF Meeting 2023). Thus, green informed consent could happen prior to a consultation as a patient-client looks for information on environmental impact about their elective treatments. The benefit to this is that IVF and other scheduled, non-urgent procedures allow for time to be informed, if the client chooses.

The other side of the initial stage of green informed consent would be urgent and emergency medicine. Here, the green informed consent process would be less relevant, in the same way that informed consent process changes when decisions must be made quickly and without dialogue. As a safeguard, health care can still be sensitive to green informed consent by making green wishes known through advance directives, DNR/DNI (Do Not Resuscitate/Do Not Intubate) orders, where applicable (Richie 2018), and, of course, making all parts of the diagnostic process itself more sustainable by therapeutic parsimony (only ordering medically necessary tests) and through life-cycle assessment of medical tools. For instance, in some cases, artificial intelligence can facilitate green medical triage and diagnostics. However, there is a trade-off with the immense amount of carbon needed for AI itself (Richie 2022a).

With these two extremes aside—elective and urgent medicine—green informed consent would begin with the medical diagnosis in hand, at the meeting between the health care professional and patient/surrogate decision makers.

Second, green informed consent offers environmental information about the diagnosis and possible medical interventions. At this point, the health care professional may provide carbon data on the procedure, or may wait to discover the patient’s values. This would occur while presenting medical options. Of course, carbon data would need to be known for this even to be a possibility. In addition to available information being a barrier to green informed consent, carbon dioxide may not be the best metric of environmental sustainability, since it acts as a proxy for only one factor of ecological importance and excludes other aspects such as waste. Moreover, carbon reduction as the end goal (for society or health care) may not be ethical as it cannot account for matters of distributive justice, both in heath care burdens, and benefits, for people, plants, animals, and ecosystems. Additionally, carbon reduction as a goal does not have normative value unless all aspects of social life—from reproduction in high carbon counties (Murtaugh and Schlax 2009), to use of fossil fuels, to global agriculture—are also quantified and reduced.

However, carbon is a qualifiable metric that has a certain cultural currency and carbon reduction is broadly understood by the public. Particularly in heath care, when there is a knowledge gap between patient and practitioner, the phrase or concept of “carbon reduction” is likely more tangible and less conceptually afar than other biotic-medical-ecological concepts like “antibiotic resistance” or “loss of biodiversity for medical breakthroughs” or “zoonotic disease transmission.” Thus, regardless of known carbon numbers, a patient might like to be aware of the fact that treating their particular condition may lead to other health complications, including those related to climate. All medical procedures do impact climate and therefore another path for green informed consent at this stage would be to address the “side effects” of any and all medical interventions—including increased risk of climate change health hazards.

While some people may object that climate change health hazards are too indirect a “side effect” to be mentioned, outside medical risks, even those that are very slight, are mentioned during the consenting process. Moreover, it is not the case that climate health hazards are a remote possibility—in 2014 the World Health Organization (WHO 2014) warned of the impacts of climate and health. Other current and predictive studies, such as one done on heat and mortality in China demonstrate that more people are affected by climate change annually (Chen et al. 2017a, b), with some researchers predicting that “by 2099, climate change could be one of the leading causes of death in the world” (Mendoza 2022). Even so, these medical externalities may not be of concern to patients just as other aspect of medical treatments, such as medical error and risk, may not be of interest and therefore need not be presented in this stage.

The first two stages of informed consent and green informed consent are rather objective. They are based on clinical diagnosis and indicated medical options with data on the benefits and drawbacks of each medical approach. As with clinical informed consent, the health care professional may have a conscientious objection to certain medical options (e.g., abortion) and it may be that there is a very high carbon treatment option that appears environmentally unsustainable. As with other dilemmas of conscious, the health care professional is required to give all clinically indicated options and to offer a referral if needed (Antommaria 2010).

In the third stage of the consent process, a dialogue occurs where the health care professional gathers information from the patient about the patient’s clinical and personal values. Here, it is the professional obligation to ask about a range of values from cost to quality of life to environemtnal impact to longevity to bodily integrity (e.g., amputations or -ectomies). Many other values like religious beliefs and impact on family and caregivers may be discussed too. These values may be informed by race, age, sex, income, and other demographics that the health care professional should be aware of (Matthew 2008).

It is neither ethical nor recommended that health care professionals impose her or his own values on the patient, so, of course green informed consent would not imply that sustainability or environmental impact must be discussed. At the same time, health care professionals may bring up irrelevant or unimportant values to the patient in an attempt to gather as much information as possible; this should not be viewed as malicious or pedantic, but rather thoughtful.

Fourth, there is a discussion of medical options with the patient, given the aforementioned values, in order to create a clinical plan. In this stage more specific information about desirable medical options is offered and explained. This tailoring of information allows for depth into the specifics of the procedure, including possible environemtnal effects, if known. These discussions always have the risk of “paternalism” in that the health care professional is more knowledgeable than the patient, so trust is important. In these conversations, the “green” aspect of informed consent would only continue if it is an expressed value of the patient. Depending on the values of the patient, the sustainability or carbon impact of the options might have little or no weight in their choice or may weigh significantly. It is not the objective of the health care professional to persuade that one value like ecology or length of treatment should factor more strongly or weakly into the patient’s choice.

Fifth and finally, the clinical plan is enacted. At this point green informed consent would function much as it did in the first stage, in that the patient has been informed, but treatments should still be as green as possible. One tactic to ensure that any medical option is sustainable would be life-cycle assessment (LCA) of individual procedures—whereby carbon calculation are done on the pipeline of the treatment and tools used during the treatment. For instance, research shows that in addition to sensible environmental strategies like divestment from fossil fuels, the carbon emissions from eyedrop pharmaceutical products used in the process of cataract surgery can be reduced by “resizing to smaller containers” (5 ml instead of 15 mL), thus ensuring less pharmaceutical waste (Tauber et al. 2019).

This strategy requires health care administrators, medical device makers, and others who contribute to the medical industry carbon footprint to green facilities, tools, and treatments through well-established strategies that enjoy broad consensus, such as efficient energy sources, recyclable materials, and streamlined medical services (Rizan et al. 2022). In some ways, then, “green” is imposed upon the patient, but not in a way that differs from buying a box of cereal that the manufacture decided should be made of recycled material or purchasing fuel that is bio-sensitive.

Given this framework, based on the existing standard of “informed consent,” green informed consent can be taught in health care professional education, taught and used in professional development and continuing education in health care institutions, and used in clinical ethics consultations. Here I am not exclusively taking the academic view of clinical ethics, since clinical ethics is not simply that which happens in the clinic, nor are clinical ethicists only those working in in health care organizations. The practice of applied clinical ethics occurs in a variety of settings anywhere people are “doing” or teaching clinical ethics, including in the classroom, the clinic, and the consultation room.

## Green informed consent in the classroom: health care professional education

Efforts to integrate sustainability into health care professional education has international support (Richie 2022b). Climate ethics in health care curriculum is—and will be—a requirement of preparing students for their role as health professionals, that is, as bioethicists in practice. To this end, the World Medical Association recognizes that students need to understand climate change in the context of health (2009). Moreover, in 2020, the International Federation of Medical Students’ Associations (IFMSA) which represents 1.3 million medical students in 133 countries drafted a “Vision of Climate Change in Medical Curricula” (2020). Currently, 129 of the 133 countries represented by the IFMSA have adopted the document (Omrani et al. 2020).

Green informed consent could be an area of topical expansion within climate change ethics in health care professional education. Already, medical schools in at least 92 countries (Omrani et al. 2020) have topics related to climate change ethics and environmental sustainability in their curriculum (Walpole et al. 2019), including the US and the UK. In the US, the American College of Physicians (ACP), “the second-largest US professional association of doctors with 148,000 members,” has officially recognized the threat of climate change and made policy recommendations in a formal Position Statement (Crowley 2019). They outline the wide range of health consequences of climate change beyond the well-known issues of air and water pollution. Moreover, the ACP acknowledges the role of the health sector in carbon emissions and indicates that “physicians and the wider health care community have a major stake in addressing climate change, not only by treating patients experiencing its health effects but also by advocating for effective climate change adaptation and mitigation policies, educating the public about potential health dangers posed by climate change, pushing for a low-carbon health care sector, researching and implementing public health strategies, and adopting lifestyle changes that limit carbon emissions and may achieve better health” (Gurevich 2020). Green informed consent supports many of these aims.

In the UK, similar efforts to place climate change ethics in health care professional education is underway. The 2009 General Medical Council (GMC)’s *Tomorrow’s Doctors* recognizes that the doctor is a “scholar, practitioner and professional” (General Medical Council 2009). Based on these three categories, Sarah Walpole, Frances Mortimer, Alice Inman, Isobel Braithwite, and Trevor Thompson proposed pathways to sustainability education in medical school curriculum in 2015. They write, “as a scholar, doctors require an understanding of how the environment and human health interact at different levels. As a practitioner, doctors must be able to apply knowledge and skills around sustainable health care in order to improve the environmental sustainability of health systems. As a professional, doctors must consider the ethical issues posed by the relationship between the environment and health” (Walpole et al. 2015). Green informed consent has the strongest connection with the “professional” category, but truly the three are mutually reinforcing.

The research of Walpole, et al. had lasting impact and in 2020, a study by the InciSioN UK Collaborative on the integration of “global health education” in medical school curriculum reported that of 20 of the 30 participating medical schools included the “future impact of climate change on health and health care systems” as a learning objective, whilst 25 listed “environmental and occupational hazards and ways to mitigate their effects” as a learning objective. The attention to environmental topics in medical school education indicates a positive and long-lasting trend, which could easily include room for education on green informed consent.

Moreover, in other health care professional education, a report from the UK indicates that “to achieve long-term improvement in the sustainability of dental services…engagement with those working in policy and education” is critical (Duane et al. 2017). Thus, pedagogical resources that align with the General Dental Council (GDC) standards “and the relationship between planetary health and human health within their practice” have been developed (Duane et al. 2019). Instructors in nursing schools also have numerous opportunities to discuss sustainability in curriculum (Lori and Madigan 2020).

Green informed consent can be placed within modules on informed consent and autonomy during foundational health care ethics and professionalism courses, with an emphasis on the “informed” aspects. Later on in the academic programs, green informed consent can be discussed in the context of stewardship of hospital and environmental resources, and in electives and selectives on public health, social determinants of health, and global health with an emphasis on the “green” aspects.

Ultimately, green informed consent should be taught in all health care education, not only in the countries that are most responsible for pollution—such as the US—but also those countries emerging in the global health care industry (Vujcich 2020; Tun 2019). Carbon emissions do not stay within national borders (Costello et al. 2009); all countries have a vested interest in educating health care professionals about sustainability, climate ethics, and the environmental impact of medical care, thus leading to changed heath care delivery practices—and changed practitioners.

## Green informed consent in the clinic: professional development and continuing education in health care institutions

In an effort to speak confidently about green informed consent, health care professionals may benefit from continuing education on the carbon impact of health care, both formally and informally. For instance, the Centre for Sustainable Health care offers formal fellowships on sustainability in specialties including nephrology; psychiatry; dentistry; public health; general practice; ophthalmology; education; anesthesia; quality improvement; and surgery (n.d.). Once educated, physicians can provide education to peers through Grand Rounds and other learning opportunities (Magdo et al. 2007).

Health care administrators can offer informal education by scheduling on-the-job training and continuing education courses on topics such as medical waste (Ghersin et al. 2020), green prescribing (Richie 2022c), environmental moral distress (Jameton 2013), and climate change health hazards, including climate anxiety (Clayton 2020).

Since numerous studies exist on the carbon footprint of specific procedures in surgery (MacNeill et al. 2017), pharmacology (Fan and Favaloro 2022), gynecology and obstetrics (Campion et al. 2012), ophthalmology (Chang and Thiel 2020), anesthesiology (Sherman et al. 2012), urology (Chen et al. 2017), dentistry (Duane et al. 2017), internal medicine (McGain et al. 2018), pathology (McAlister et al. 2020), and single use and disposable instrument (Eckelman et al. 2012)—and many more are being performed yearly—easy of data will increase. Thus, during consultations, if the carbon footprint or resource use of a treatment plan is known it should be disclosed. In fact, in green informed consent, a health care provider might be withholding information if she did not provide the patient with environmental data.

To facilitate these discussions, the environmental impact of health care can be printed and displayed—such as posters on the walls of doctor offices or in leaflets health care providers could refer to during patient consultations. There are number of easy-to-read charts on carbon data and other aspects of sustainable health care which can be adopted or modified from already existing literature. These charts may list the specific carbon emissions of individual procedures, such as the one in Duane et al. (2017), or they may compare two medical options side by side with columns indicating the relative carbon impact (Campion et al. 2012). Also, carbon emissions may not be the only factor identified in these environmental charts. Campion et al. (2015) include many environmental factors like ozone depletion, global warming (measured in CO_2_), smog, acidification, eutrophication, carcinogenics, non-carcinogenics, respiratory effects, ecotoxicity, and cumulative energy demands. Another option to make green informed consent simple in the clinic might be color-coded labelling of products, like pre-sterilized medical tool, that display the environmental “rating” of a product from A to E (with A being the “best”). This is similar to markings widely used on food packaging and products like lightbulbs.

Even if this data is unknown, consenting the patient about the side effects of their care—which may include climate change health hazards—would fulfil the obligation of the physician for truth-telling and give the patient relevant information with which to make a treatment choice.

While patients should be aware that their health care emits carbon, contributes to climate change, and may affect them through climate change health hazards, they should not bear the burden of research into the environmental effects of health care, as they are often in the vulnerable position of needing or seeking medical treatments and are not required to be experts in either medical care options or carbon emissions of health care. Therefore, patients may expect—and be prepared— for health care providers to disclose the environmental effect of various procedures during green informed consent, which is already the standard in some health care facilities (King and Brown 2009).

## Green informed consent in the consultation room: clinical ethics

One of the most visible ways that bioethicists contributes to the ethical structure and functioning of health care institutions in a number of countries is through clinical ethics consultations (Hurst et al. 2007; Fox et al. 2007). Clinical ethics consultations may be requested by a patient, health care provider, or patient advocate and seek to align health care delivery with the patient’s values. Clinical ethics dovetails with biomedical ethics in the commitment to ethical health care, but rather than drawing on Tom Beauchamp and James Childress’ *Principles of Biomedical Ethics* alone (1979), clinical ethics also utilizes an ethical methodology developed by Albert R. Jonsen, Mark Siegler, and William J. Winslade (2015), centered on four discrete topics for organizing ethical reasoning: medical indications, patient preferences, quality of life, and contextual features.

“Contextual features” includes law, public health, personal and national finances, and institutional policy. The environmental impact of medical care fits best under this category (Richie 2018) and supports the practice of green informed consent in clinical ethics consultations. Oftentimes, consults are called in relation to futile end-of-life care, moral distress, length of days in the hospital with little improvement or chance of meaningful recovery, and other difficult decisions.

If a consult is called, green informed consent during clinical ethics consultations can be further supported by already available resources. For instance, end-of-life care is very challenging for patients and family, but the “My Way Cards for Natural Dying” informative cards were developed by Caring Advocates (2014) to support the natural death movement, which Carol Taylor and Robert Barnet describe as “not depend(ing) on high tech medicine” (2014). The “My Way Cards for Natural Dying” can be viewed online and provide a detailed guide for advanced directive decisions in the context of green informed consent. Clinical ethics can, of course, utilize green informed consent in other ways related to contextual features.

While learning the patient’s goals and preferences related to their care—which may or may not include the attendant environmental impact—can facilitate true consent on important medical issues, at the same time, sometimes hospitals policies are based on economics or workforce availability and these may trump patient wishes for non-clinically indicated treatments. Therefore, we ought not exclude the possibility that in addition to a clinical basis for discontinuing treatment or moving a patient to a different facility, there might be other factors such as cost, personnel, and environmental impact factored into the equation. However, green informed consent may be especially poignant during end-of-life care as all humans will face mortality, as we move “from birth to earth.”

## Conclusion: greening beyond health care

The environmental impact of health care delivery has been underconsidered, in part, because of the mistaken impression that all available health care delivery is medically necessary and therefore carbon emissions are morally irrelevant or insignificant. However, not all health care is medically necessary or clinically indicated. Much has been written about medical waste, the harm of overtreating, and medicalization (Barratt and McGain 2021). Since clinical necessity is a prerequisite for clinical medicine, in addition to green informed consent for medically necessary procedures, aspects of health care delivery outside that scope can be immediately targeted for reduction with environmental impact as an additional reason alongside sound economic and biomedical arguments (Edoka and Stacey 2020). This would support a greener health care system overall, perhaps “freeing up carbon” for other medically indicated treatments.

There are a number of ways to reduce non-urgent or non-medically necessary procedures. James Childress and Tom Beauchamp have advocated for a multi-tiered approach to health care, whereby basic standards of care would include “public health measures and preventive care, primary care, acute care, and special social services for those with disabilities” and a second or third level that provides services beyond “basic care” (1994, p. 16). The second or third levels of care would therefore be an initial place to begin greening health care, which would also adhere to ethical health care allocation, broadly endorsed by policymakers. These strategies are in effect in a number of countries.

Ian M. Seppelt reports that in Australia, “elective admissions (mainly elective surgery) rationing is achieved by waiting, at times up to a year for non-urgent surgery, and patients are treated by a hospital-appointed doctor, rather than necessarily the doctor of their choice” (2013, p. 4). Again, there is an *additional*, but not exclusive, environmental rationale for this in addition to those already discussed, such as economics and pressure on health care delivery.

As discussed above, green informed consent, life-cycle assessment, and allocation can make health care more sustainable. Of course, outside of health care priority setting is necessary. Society might endorse the elimination of certain high-carbon activities altogether. Surely the entirety of health care, as a core human right and value, would not face this assessment, whereas all of fast fashion or private transportation might. But even fashion and transportation are situated with other human needs or capabilities—for protective outwear and mobility (Nussbaum 1997). Therefore, a number of researchers have proposed a “donut framework” to balance the needs of humans, which require carbon, with the bounds of other global priorities (Raworth 2017) such as a clean and healthy ecosystem which sustains all of life. In the donut, carbon is expended in a thoughtful and ethical way that does not impose or endanger the very systems we depend on.

Environmental ethics is more frequently an interlocutor with social ethics and health care. This fits with the desires and moral compass of health care and health care professionals. Research from a dissertation on the carbon impact of mental health care found that health care professionals do want discuss the environmental impact of their profession but that the top two potential barriers to “clinician engagement with sustainability are limited staff time and poor support from management” (Maughan 2015, Fig. 13). Currently, studies are being done on how to overcome this (Hantel 2022–2024) and streamline concepts related to green informed consent.

As proposed here, doctors and health care professionals must connect their health care delivery with carbon emissions and minimize resource use when possible as a part of their obligation to do no harm (the principle of non-maleficence). Patients receiving treatments should be aware that their health care in the clinic may lead to more medical treatments later, should they encounter climate change health hazards. Health care administrators and personnel should be educated about the risks of readmission due to climate change health hazards.

Green informed consent, and the many other initiatives for resource conservation in health care (Healthier Hospitals Initiative 2017), must be part of health care delivery. The role of clinical ethics—broadly understood—must include advocacy for environmental sustainability. To be sure, green informed consent does not have to be placed within *ethics* curriculum, *ethics* training in the clinic, or *clinical ethics* consultations since climate change health hazards and the carbon emissions of health care delivery are more than ethical issues; they impact all areas of medicine. Indeed, the impetus for environmental sustainability must be part of what it means to heal; not only the body but also the *bios* in which our bodies live (Potter 1982; Kemple 2020).
